# B7-H3-targeted CAR T cell activity is enhanced by radiotherapy in solid cancers

**DOI:** 10.3389/fonc.2023.1193963

**Published:** 2023-07-07

**Authors:** Marco Ventin, Giulia Cattaneo, Luke Maggs, Jingyu Jia, Shahrzad Arya, Soldano Ferrone, Xinhui Wang, Cristina R. Ferrone

**Affiliations:** ^1^ Division of Gastrointestinal and Oncologic Surgery, Department of Surgery, Massachusetts General Hospital, Harvard Medical School, Boston, MA, United States; ^2^ Department of Orthopaedic Surgery, Massachusetts General Hospital, Harvard Medical School, Boston, MA, United States; ^3^ Department of Surgery, Cedars Sinai Medical Center, Los Angeles, CA, United States

**Keywords:** immunotherapy, radiotherapy, solid cancer, combinatorial therapy, B7-H3 CAR T

## Abstract

Adoptive cell therapy utilizing T cells genetically modified to express a chimeric antigen receptor (CAR) has demonstrated promising clinical results in hematological malignancies. However, solid cancers have not seen a similar success due to multiple obstacles. Investigating these escape mechanisms and designing strategies to counteract such limitations is crucial and timely. Growing evidence in the literature supports the hypothesis that radiotherapy has the potential to enhance the susceptibility of solid tumors to CAR T cell therapy, by overcoming mechanisms of resistance. Radiation treatment can increase the susceptibility of different types of solid cancers (TNBC, HNSCC, PDAC) to B7-H3 CAR T cell-mediated eradication. Multiple mechanisms, including reduced cancer cell proliferation, upregulation of the targeted antigen, modulation of apoptotic molecules may contribute to this signal. The information in the literature and the results we describesupport the ability of radiotherapy to improve the efficacy of CAR T cell therapy in solid tumors.

## Introduction

T lymphocytes genetically modified to express a tumor antigen (TA)-specific chimeric antigen receptor (CAR) represent a very promising immunotherapeutic strategy for the treatment of solid and hematological malignancies refractory to currently available therapies. CARs are recombinant proteins composed of an extracellular recognition domain, linked to an intracellular T cell activation domain through a spacer/hinge region and a CD28 transmembrane portion. The antigen-binding ectodomain derives from a single-chain variable fragment (scFv) of a monoclonal antibody and mediates TA recognition and subsequent activation of the effector mechanism (1). The endodomain is comprised of a CD3ζ signaling chain alone (first-generation CARs) or linked with one (second generation) or two (third generation) co-stimulatory domains, which often include CD28, 4-1BB, ICOS and OX40 (2, 3). Fourth generation CAR T cells (T cell redirected for universal cytokine-mediated killing, TRUCKs), include a ‘payload’, for instance, an immune-stimulatory cytokine, which is released following the interaction with the target. This strategy augments CAR T cell activation and stimulates the innate immune response and its recruitment to the tumor site (4). Fifth- generation CARs contain an additional truncated intracellular domain of cytokine receptors (e.g., IL-2R chain fragment) which, by binding to transcription factors such as STAT-3/5, promotes CAR T cell activation, generation of memory T cells and stimulation of the immune system (5). The CAR-mediated recognition of the TA does not require its processing and presentation by the HLA class I complex (6). Therefore, CAR T cell-mediated elimination of cancer cells is not negatively affected by absent or defective expression and/or function of HLA class I and antigen processing machinery (APM) components. The latter is one of the most common escape mechanisms utilized by cancer cells to evade T cell-mediated immune responses and is associated with tumor progression in many malignancies (7). The impressive clinical responses obtained with hematological malignancies have led to approval of four CD19- and two BCMA-targeted CAR T cell products by the US Food and Drug Administration (FDA) for the treatment of patients with relapsed or refractory B-cell malignancies and multiple myeloma (8).

The great successes achieved with hematological malignancies, has not been seen with CAR T cell therapy for solid tumors. Indeed, the results generated from the ongoing clinical trial investigating the efficacy of CAR T cells as monotherapy or in combination with other conventional treatments are disappointing due to limited therapeutic efficacy, generally low cure rates and lack of durable clinical benefits (9).

In this review we summarize some of the mechanisms associated with failure of CAR T cell therapy in solid tumors, as well as addressing strategies which have been adopted to overcome such limitations, with a particular focus on the use of CAR T cells in combination with radiotherapy.

## CAR T cells for the treatment of solid cancers, a challenging landscape

CAR T cells as a monotherapy for solid tumors such as breast, pancreatic, renal, colorectal, prostate, ovarian and lung cancers, brain tumors, hepatocellular carcinoma have demonstrated limited efficacy (10). Identification and characterization of the mechanisms underlying the poor efficacy of CAR T cells with solid cancers are of paramount importance for the design of novel combinatorial strategies and clinical trials (11). Limited CAR T cell trafficking and infiltration of the tumor site, inadequate persistence *in vivo*, as well as the presence of a highly immunosuppressive tumor microenvironment (TME) with target TA loss and heterogeneity, are among the major obstacles for CAR T cell therapy in solid tumors (12).

### Immunosuppressive tumor microenvironment

A major barrier to CAR T cell therapy is represented by the immunosuppressive TME, which limits CAR T cell infiltration, activation, and subsequent elimination of cancer cells (13). The complexity of the TME is in part due to the presence of different types of stromal cells, such as cancer-associated fibroblasts (CAFs), that sustain cancer cell proliferation, migration, and de-differentiation (14), promote the formation of a dense extracellular matrix, a physical barrier for CAR T cell infiltration, and contribute to the abnormal growth of the tumor-associated vasculature (15). Additionally, the TME is highly infiltrated with an immune cell population endowed with immune suppressive activity, such as myeloid-derived suppressor cells (MDSCs), regulatory T cells (Tregs), tumor-associated macrophages (TAMs) (16), and enriched by immunosuppressive factors, such as adenosine, galectin-1, the type 1 and type 2 indoleamine, 2,3 dioxygenase (IDO1 and IDO2) enzyme, transforming growth factor-beta (TGF-β) and vascular endothelial growth factor (VEGF) (17).

Hypoxia is another hallmark of the TME which strongly contributes to the establishment of an hostile microenvironment. Hypoxia induces oxidative stress and metabolic alterations that ultimately result in TME acidification and suppression of T cell activation, proliferation and cytotoxicity (18). Furthermore, hypoxic zones are enriched with MDSCs, TAMs and Treg cells, which strongly suppress the immune response by inducing T cell inhibition and anergy (19).

CAR T cell function is also hampered by the interaction between the immune checkpoint PD-1 expressed on T cells and its ligands PD-L1 and PD-L2 expressed on cancer cells and other immune components of the TME. It is well established that the PD-1/PD-L1, PD-L2 axis leads to T cell exhaustion (20). Hypoxia-inducible factor-1α (HIF-1α) induces the expression of PD-L1 on breast cancer and prostate cancer cells (21). In addition, PD-L1 is expressed under hypoxic conditions on immune cells such as dendritic cells, MDSCs and macrophages (22). Several other immunosuppressive pathways may be involved in cancer cell escape from CAR T cells, such as CTLA-4 and its ligands CD80/86, galectin 9 and TIM-3, the immune checkpoints TIGIT and LAG-3 with their respective ligands CD155/CD112 and LSECtin as well as the V-domain Ig suppressor of T cell activation (VISTA), which binds to VSIG-3 molecule (20).

### Target tumor antigen selection

Besides the limitations described above, selection of the “right” TA is undoubtedly a critical step for the success of CAR T cell therapies. CAR T cells for various antigens on solid tumors, such as HER2, MUC1, mesothelin, CEA, ROR1, GD2, EGFR, are currently being investigated (23). Identifying TAs uniquely expressed on cancer cells, without being present on normal tissues to decrease the risk of “on-target, off-tumor” toxicity is challenging (24–27). Furthermore, most TAs used as CAR T cell targets, are characterized by a high intra- and inter-lesion heterogeneity. Such heterogeneity provides cancer cells an escape mechanism potentially leading to the generation of escape variants resistant to CAR T cell-mediated recognition and killing because of the lack of the targeted TA expression. This issue has been reported with many TAs including, but not limited to, mesothelin in non-small cell lung cancer (NSCLC) and pancreatic cancer (28, 29), HER2 in NSCLC (30), and EGFRvIII in glioblastoma (GBM) (31). Another immune escape mechanism is the complete or partial loss of the CAR-targeted epitope and/or TA. Under the pressure of CAR T cells, cancer cells can downregulate the expression level of the targeted epitope and/or TA below the threshold necessary for CAR T cell activation (32). Furthermore, alternative splicing and mutations can lead to the expression of a TA that cannot be recognized by the CAR construct used. The latter escape mechanism has been associated with the development of resistance to CD19 CAR T cells in patients with acute lymphoblastic leukemia (33).

### B7-H3 as ideal target TA for CAR T cell-based immunotherapy

B7-H3, the membrane protein also known as CD276, is a CAR T cell targeted TA with significant potential. B7-H3 is a member of the human B7 family ligands involved in mechanisms of cancer invasion and migration, tumor angiogenesis, and is associated with both co-stimulatory and co-inhibitory T cell functions (34). B7-H3 is aberrantly and homogeneously expressed across many types of solid tumors, with a very limited distribution on normal human tissues, with the exception of gastric epithelial cells, salivary glands and adrenal glands at a level likely to be lower than that required for the activation of B7-H3 CAR T cells (35). B7-H3 expression correlates with poor prognosis in several cancers, such as intrahepatic cholangiocarcinoma (ICC), colorectal cancer, and prostate cancer (35).

Notably, B7-H3 is highly expressed on a subpopulation of cancer initiating cells (CICs) in different types of solid cancers (36, 37). According to the cancer stem cell theory, these cells are responsible for tumor heterogeneity, disease progression, metastatic spread and resistance to conventional therapies, especially chemotherapy and radiotherapy (38). Therefore, the application of therapies able to target and eliminate this subpopulation is crucial to achieve complete tumor eradication and prevent relapse or recurrence. CICs are particularly adept to T cell-based immunotherapy resistance due to their low immunogenicity and the defective expression and/or function of HLA class I APM molecules (39). In this regard, CAR T cell-based immunotherapy has the potential to overcome these limitations, by effectively targeting and eradicating CICs. CAR T cells redirected against specific TAs expressed by CICs, such as ALDH, CD133, CD90, and EpCAM are currently under investigation in clinical trials (37, 40).

In our previous studies, we have demonstrated that B7-H3 is highly expressed, with limited heterogeneity, on both differentiated and CICs in head and neck squamous cell cancer (HNSCC), triple negative breast cancer (TNBC), prostate cancer (PC), intrahepatic cholangiocarcinoma (ICC) and pancreatic ductal adenocarcinoma (PDAC) cell lines and surgically resected specimens (34, 35, 37, 41). Thus B7-H3 CAR T cell therapy represents potentially effective strategy to target both differentiated cancer cells and CICs, as well as overcome the resistance mechanisms associated with CICs in solid tumors.

## Strategies to enhance CAR T cell efficacy with solid cancers

Several efforts have been made to improve the therapeutic safety and efficacy of CAR T cells in solid tumors (42). The preclinical application of T cells genetically modified to co-express a TA-specific CAR with chemokine receptors, including CXCR1 or CXCR2 has demonstrated an improvement in cell trafficking and antitumor activity in several types of solid cancers (43, 44). Innovative engineering approaches have been used to address the limitations of antigen escape and tumor heterogeneity by developing CARs able to simultaneously target multiple antigens. These strategies include the design of multi-antigen-targeted CAR T cells, based on the engineering of T cells to co-express two (dual CAR T) or three (trivalent CAR T) individual CARs specific for different TAs, or to express a single CAR comprised of two distinct antigen-binding domains connected in tandem (tandem CAR T) (45). Additional systems rely on the application of CAR T cells engineered to co-express and secrete bi-specific T cell engagers (BiTEs) (46). To enhance the specificity, safety and programmability of CAR T cells, universal CARs for which adapter molecules or immunoglobulin Fc motifs are used as ligands to enable the targeting of multiple TAs (split, universal and programmable (SUPRA)) have been designed (47).

Other approaches are focused on improving the ability of CAR T cells to penetrate physical barriers to enter the complex TME, prolong their *in vivo* persistence, and counteract inhibitory signals which ultimately lead to CAR T cell exhaustion (48).

Therapeutic strategies combining CAR T cells with other treatments, such as small molecule inhibitors and Immune Checkpoint Inhibitors (ICI), are currently being investigated in preclinical studies and clinical trials (49).

### Radiotherapy to increase the efficacy of CAR T cell-based immunotherapy with solid tumors

Our group’s efforts focus on mechanisms to improve the efficacy of CAR T cells against different types of solid cancers, including HNSCC, TNBC, PC and PDAC, all aggressive malignancies with a dismal prognosis and inadequate available therapies. Adoptive immunotherapy using B7-H3-targeted CAR T cells represents an attractive and promising approach to address this unmet clinical need.

Preclinical and clinical evidence supports the hypothesis that radiotherapy can synergize with CAR T cell therapy for the treatment of various types of solid cancers (37, 50). Radiotherapy enhances cancer cell susceptibility to CAR T cell-mediated eradication through multiple mechanisms, including its ability to inhibit and control cancer cell growth, metastatic spread, and proliferation by inducing DNA damage to malignant cells (51, 52).

**Figure 1:**
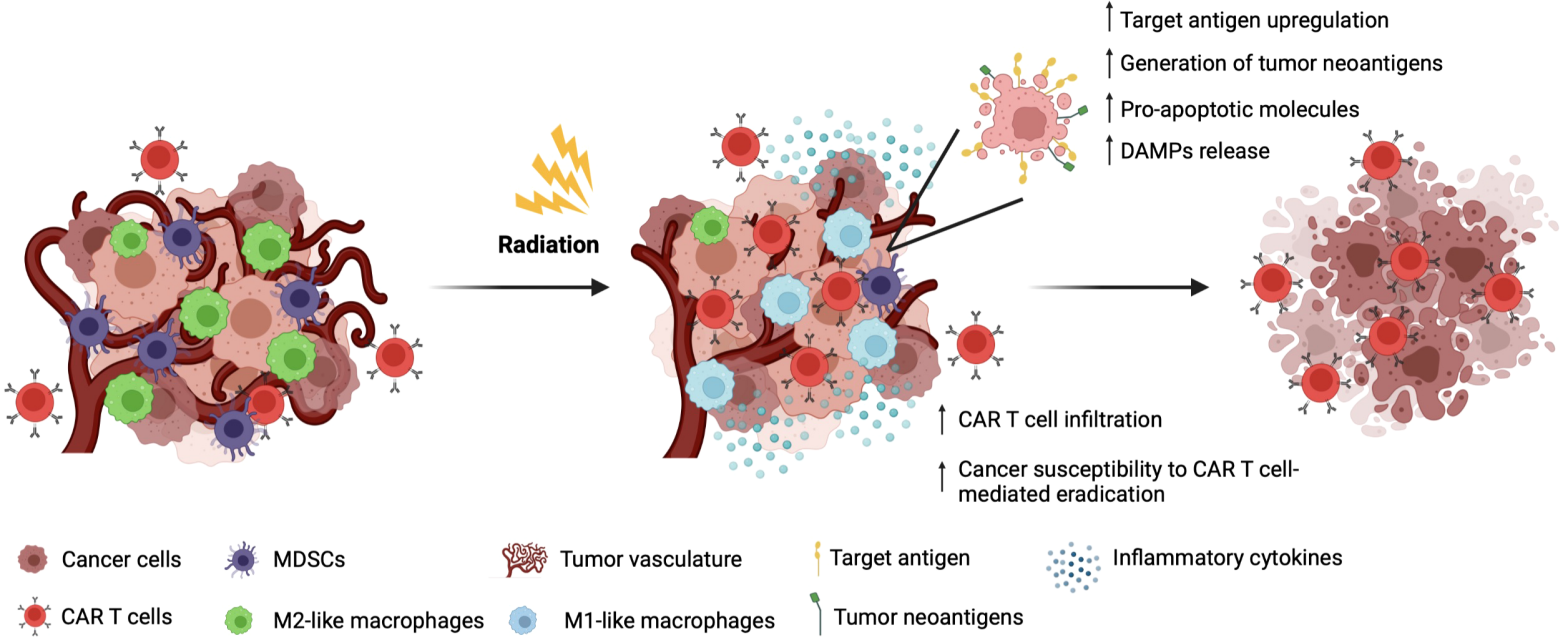
Radiotherapy to increase the efficacy of CAR T cell-based immunotherapy with solid tumors.

Radiotherapy has been proven to act as a TME-modulating therapy capable of rendering the TME less hostile to CAR T cell trafficking and infiltration, by normalizating aberrant tumor vasculature, and upregulating the expression of adhesion molecules on the endothelium of tumor vessels, both mechanisms which facilitate CAR T cell extravasation and local expansion (53, 54). Radiotherapy also has the potential to reverse the immunosuppressive status of the TME by reducing the frequency of immunosuppressive M2-like macrophages and MDSC within the tumor bulk (51). Notably, a decreased level of circulating MDSCs has been observed in lung cancer patients following conventional fractionated radiotherapy treatment (55) (**Figure 1**).

Through the activation of the STING pathway, radiotherapy stimulates the upregulation of interferon-related genes in cancer cells, which leads to the release of pro-inflammatory cytokines, such as type I IFNs (IFN-α and IFN-β). This inflammatory status facilitates dendritic cell maturation and effector T cell recruitment and activation (56).

Furthermore, radiotherapy may increase TA presentation and expression (37, 57), and induce an upregulation of the proapoptotic protein Bax and downregulation of the antiapoptotic protein Bcl-XL, favoring the balance towards apoptosis (58), and potentially making cancer cells more susceptible to CAR T cell-mediated lysis.

Radiotherapy is limited in the setting of metastatic disease as it is a local rather than a systemic treatment. However, radiotherapy can elicit systemic immunomodulatory effects by inducing an abscopal effect, defined as ‘the ability to induce a clinical response at a distant sites’ (59). Based on this concept, several clinical studies are exploring the synergism between radiotherapy and immunotherapy (60). Interestingly, it has been shown that fractionated but not single dose radiotherapy was capable of synergizing with ICI inducing the STING pathway activation and promoting an abscopal immune response (61).

Beneficial effects on tumor immunity induced by radiotherapy include the generation of tumor neoantigens and the induction of immunogenic cell death (ICD), mechanisms which activate an immune response which may synergize with CAR T cell-mediated antitumor activity (62).

In our studies, we investigated whether the treatment of TNBC, HNSCC and PDAC cells with radiotherapy could enhance their susceptibility to B7-H3 CAR T cell-mediated eradication *in vitro*.

### Radiotherapy increases the susceptibility of cancer cells to B7-H3 CAR T cell- mediated eradication *in vitro*

**Figure 2:**
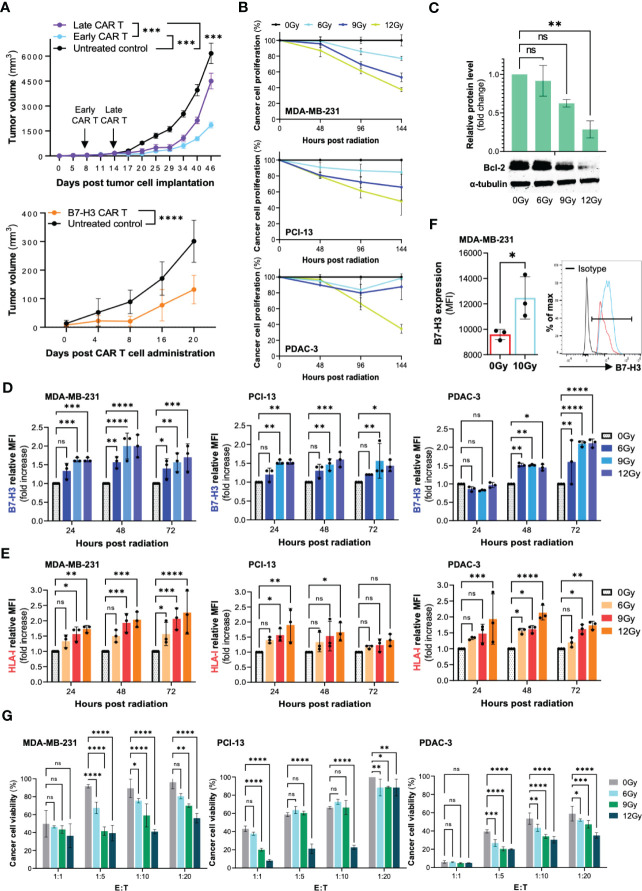
Radiation can increase the susceptibility of cancer cells to B7-H3 CAR T cell-mediated elimination *in vitro* through multiple mechanisms. The human TNBC (MDA-MB-231), HNSCC (PCI-13), and PDAC (PDAC-3) cell lines were utilized to perform the outlined experiments. **(A)** The TNBC cell line MDA-MB-231 was orthotopically grafted in the mammary fat pad of NSG mice (2x10^6 cells/mouse). B7-H3 CAR T cells (5x10^6 CAR T cells/mouse) were administered by tail vein to groups early CAR T and late CAR T (8 and 14 days following tumor cell engraftment, respectively). The HNSCC cell line PCI-13 was subcutaneously grafted in the right flank of NSG mice (2x10^6 cells/mouse). B7-H3 CAR T cells (5x10^6 CAR T cells/mouse) were administered by tail vein injection. Untreated groups of mice were utilized as a control. Tumor volume was monitored by caliper measurement. **(B)** Cancer cells (MDA-MB-231, PCI-13 and PDAC-3) were seeded in a 96-well tissue culture plate (5,000 cells/well) and treated with a total radiation dose of 6Gy, 9Gy, and 12Gy administered in daily fractions of 2Gy, 3Gy and 4Gy, respectively. Untreated cells (0Gy) were included as controls. The extent of cancer cell proliferation was monitored for 6 days by performing MTT assay every 48 hours. **(C)** MDA-MB-231, PCI-13 and PDAC-3 cells were treated with radiation, as described. Following a 48-hour incubation, cancer cells were collected, and the expression level of the apoptotic protein Bcl-2 was assessed by Western Blot. **(D, E)** MDA-MB-231, PCI-13 and PDAC-3 cells were seeded in a 6-well tissue culture plate (100,000 cells/well) and treated with radiation, as described. The expression level of B7-H3 and HLA class I on cancer cells was assessed by flow cytometry at different timepoints (24 hours, 48 hours, 72 hours) following radiation treatment. The mouse anti-human monoclonal antibodies (mAb) 376.96 and TP2.25, specific for B7-H3 and HLA class I respectively, were utilized for the analysis. Changes in the expression level are shown as fold increase of the Mean Fluorescence Intensity (MFI) compared to the untreated control. **(F)** The TNBC cell line MDA-MB-231 was orthotopically grafted in the mammary fat pad of NSG mice (5x10^6 cells/mouse). When tumors reached 0.5cm in diameter, a group of mice (n=3) was treated with a radiation dose of 10Gy, which was locally administered to the tumor site as a single dose. A group of mice (n=3) was left untreated as control. Two days following the administration of radiation, tumors were collected, enzymatically digested, and analyzed by flow cytometry for B7-H3 expression. **(G)** The MDA-MB-231, PCI-13, and PDAC-3 cell lines were plated in a 6-well tissue culture plate (100,000 cells/well) and treated with fractionated radiation at the indicated total dose. Following a 48-hour incubation, cancer cells were collected, counted and cocultured with B7-H3 CAR T cells at different effector to target (E:T) ratios (1:1, 1:5, 1:10, 1:20) for 3 days. At the end of the incubation period, cancer cell viability was assessed by MTT assay. All the described experiments were performed three independent times for each cell line, and results are shown as mean ± SD. *, ≤0.05; **, ≤0.01; ***, ≤0.001; ****; ≤0.0001.

Our preliminary studies have shown a limited efficacy of B7-H3 CAR T cells as monotherapy in murine models of HNSCC and TNBC. As shown in **Figure 2A**, B7-H3 CAR T cells demonstrated the ability to control tumor growth, but not to eradicate HNSCC tumors subcutaneously grafted in NSG mice. Similar results were observed in an orthotopic model of TNBC. Furthermore, in the latter model, we observed that B7-H3 CAR T cells were more effective in controlling tumor growth when given early to mice harboring tumors of smaller size. These findings provided us with the rationale to explore the possibility to overcome this lack of efficacy and increase CAR T cell activity by pre-treating cancer cells with radiotherapy.

To effectively combine radiotherapy with CAR T cells, we have used relatively low total doses of radiotherapy (6Gy, 9Gy, 12Gy) prior to CAR T cell administration. In our preliminary results we observed that radiotherapy significantly limited cancer cell proliferation (**Figure 2B**). We found that radiotherapy caused some direct apoptosis (as showed by the decreased level of the anti-apoptotic protein Bcl-2) without completely eradicating the cancer cell population (**Figure 2C**). In addition, we observed a dose and time-dependent increase in the expression level of B7-H3 and HLA-I on TNBC, HNSCC and PDAC cells following radiotherapy treatment (**Figures 2D, E**). A significant upregulation of B7-H3 expression induced by radiation was also observed on TNBC tumors orthotopically grafted in NSG mice (**Figure 2F**).

To confirm the validity of our hypothesis, TNBC, HNSCC and PDAC cell lines were treated with increasing doses of radiotherapy (6Gy, 9Gy, 12Gy) administered with fractionated schedule and then cocultured with B7-H3 CAR T cells at different effector to target cell (E:T) ratios. Irradiated cancer cells were eradicated to a significantly higher extent compared to the untreated control in a dose-dependent manner. Notably, this effect turned out to be more enhanced when CAR T cells were tested at low E:T ratios, a challenging condition for CAR T cells (**Figure 2G**).

Altogether, these preliminary results support the hypothesis that radiotherapy could be a valid strategy to improve the antitumor activity of CAR T cells with solid tumors.

## Perspective

The design of novel combinatorial therapies is of paramount importance to overcome the failure of CAR T cell therapy with solid tumors. To this end, radiotherapy represents a valid strategy capable of addressing most of the limitations associated with the limited efficacy of CAR T cells with solid tumors, especially by controlling tumor growth, altering the immunosuppressive nature of the TME, and making cancer cells more susceptible to the cytotoxic effect mediated by CAR T cells. One potential mechanism through which this may occur is by increasing or restoring the expression of the B7-H3 TA on cancer cells as there is a direct correlation between the level of TA expression and CAR T cell activity (37, 63). Clearly, radiotherapy is not specific in its effects on B7-H3, and modulation of multiple protein expression occurs, such as the promotion of proapoptotic molecules which may lower the threshold of activation required for CAR T cell-mediated killing.

It is of note that modulation of some inhibitory molecules on cancer cells, such as the upregulation of PD-L1, may have a negative impact (64), by promoting CAR T cell exhaustion. Therefore, further combination with ICI may be necessary to optimize this strategy.

In conclusion, despite several questions remain to be addressed (optimization of dose and fractionation applicability to a broad range of cancer types), the information available in the literature and the preliminary results we have described provide the rationale to further investigate the positive impact of radiotherapy on CAR T cell immunotherapy for future preclinical and clinical applications.

## Data availability statement

The raw data supporting the conclusions of this article will be made available by the authors, without undue reservation.

## Ethics statement

The animal study was reviewed and approved by Institutional Animal Care and Use Committee (IACUC) of Massachusetts General Hospital.

## Author contributions

Concept and design: SF. Acquisition, analysis, or interpretation of data: MV, GC, LM. Drafting of the manuscript: MV, GC, LM, XW. Critical revision of the manuscript for important intellectual content: All authors. Obtained funding: SF, XW. Supervision: SF, CRF, XW. All authors contributed to the article and approved the submitted version.
